# Serum small RNA profiling identifies prognostic biomarkers for sepsis mortality prediction

**DOI:** 10.3389/fmed.2025.1665726

**Published:** 2025-09-29

**Authors:** Yiming Wang, Jinzhong Dong, Houxing Wang, Jie Li, Shan Zhang, Jianhua Zhu, Hao Wang, Guodong Chen

**Affiliations:** ^1^Department of Critical Care Medicine, The First Affiliated Hospital of Ningbo University, Ningbo, Zhejiang, China; ^2^Institute of Environmental Medicine, Zhejiang University School of Public Health, Hangzhou, Zhejiang, China; ^3^Department of Critical Care Medicine, The Second Affiliated Hospital, Zhejiang University School of Medicine, Hangzhou, Zhejiang, China; ^4^Department of Laboratory Test, Yinzhou No. 2 Hospital, Ningbo, Zhejiang, China; ^5^Department of Emergency Intensive Care Unit, Yinzhou No. 2 Hospital, Ningbo, Zhejiang, China

**Keywords:** sepsis, tRNA-derived small RNAs, microRNAs, biomarkers, mortality prediction, ROC analysis

## Abstract

**Background:**

Sepsis remains a leading cause of mortality in critically ill patients, with current prognostic tools showing limited accuracy for outcome prediction. While traditional clinical parameters and inflammatory biomarkers provide some prognostic information, there is an urgent need for novel molecular biomarkers that can accurately predict sepsis outcomes to guide clinical decision-making and therapeutic interventions. Circulating small RNAs, including tRNA-derived small RNAs (tsRNAs) and microRNAs (miRNAs), have emerged as potential biomarkers due to their stability in circulation and regulatory roles in immune responses and inflammatory processes.

**Methods:**

This study enrolled 26 sepsis patients admitted to the intensive care unit, who were stratified into recovery (*n* = 17) and death (*n* = 9) groups based on clinical outcomes. Comprehensive clinical parameters including demographic characteristics, severity scores, inflammatory markers, organ function indicators, metabolic parameters, and acid–base balance were analyzed. Serum samples underwent optimized small RNA profiling using high-throughput sequencing with de-modification protocols to enhance tsRNA and miRNA detection. Differential expression analysis was performed to identify outcome-associated small RNAs, and receiver operating characteristic curve analysis was conducted to evaluate diagnostic performance of individual biomarkers and combined panels.

**Results:**

Traditional clinical parameters showed limited prognostic value, with only specific markers including SOFA scores, procalcitonin, interferon-*γ*, glucose levels, and acid–base parameters demonstrating significant associations with outcomes. Small RNA profiling revealed 22 differentially expressed tsRNAs (12 downregulated, 10 upregulated) and 5 differentially expressed miRNAs (3 downregulated, 2 upregulated) in the death group compared to the recovery group. Individual biomarkers showed substantial discriminatory power, with top-performing tsRNAs achieving AUCs of 0.827–0.837 and miRNAs reaching AUCs of 0.797–0.850. Notably, combined biomarker panels demonstrated exceptional diagnostic performance, with the tsRNA signature achieving an AUC of 0.967 and the miRNA panel reaching an AUC of 0.902.

**Conclusion:**

This study identifies circulating small RNAs as highly promising novel biomarkers for sepsis outcome prediction, substantially outperforming traditional clinical parameters. The exceptional diagnostic accuracy of combined tsRNA and miRNA signatures suggests significant potential for clinical translation to improve sepsis prognosis and patient stratification. These findings provide a foundation for developing molecular-based prognostic tools that could enhance sepsis management and guide therapeutic decision-making in critically ill patients.

## Introduction

Sepsis, defined as a life-threatening organ dysfunction caused by a dysregulated host response to infection, represents one of the most pressing global health challenges (1). Recent estimates suggest that sepsis affects approximately 49–50 million people globally each year, with mortality rates remaining substantially high despite advances in critical care (2, 3). The management of sepsis remains challenging, particularly in accurate prognostication for individual patients (4, 5). Early identification of patients at high risk of mortality is crucial for timely intervention, resource allocation, and prognostic counseling (6). This underscores the urgent need for accurate and reliable biomarkers that can predict sepsis outcomes and guide clinical decision-making in the intensive care setting.

Current prognostic tools for sepsis rely primarily on clinical scoring systems and conventional biomarkers. However, their predictive accuracy remains suboptimal for individualized patient management (1, 7, 8). The Sequential Organ Failure Assessment (SOFA) score and Acute Physiology and Chronic Health Evaluation II (APACHE II) score are widely used severity scoring systems that incorporate physiological parameters and organ dysfunction indicators, but their discriminatory power for mortality prediction varies considerably across different populations (9–11). Moreover, traditional inflammatory biomarkers such as C-reactive protein (CRP), procalcitonin (PCT), and pro-inflammatory cytokines including interleukin-6 (IL-6) and tumor necrosis factor-*α* (TNF-α) have shown promise in sepsis diagnosis and monitoring, but their prognostic value for predicting clinical outcomes remains inconsistent (12–14). The limitations of existing prognostic tools underscore the critical need for novel molecular biomarkers that can provide superior predictive accuracy and capture the complex pathophysiological processes underlying sepsis progression and patient outcomes.

Small non-coding RNAs have emerged as promising molecular biomarkers due to their regulatory roles in gene expression, immune responses, and cellular stress responses, with particular relevance to sepsis pathophysiology (15, 16). Among these, tRNA-derived small RNAs (tsRNAs) represent a recently discovered class of regulatory molecules that are generated through specific cleavage of transfer RNAs under stress conditions and play important roles in cellular adaptation, immune regulation, and inflammatory responses (17–19). Based on their cleavage sites and mapping positions relative to mature tRNA sequences, tsRNAs can be classified into several subtypes: 5’-tRNA halves (5’-tiRNAs) and 3’-tRNA halves (3’-tiRNAs) produced by angiogenin cleavage at the anticodon loop, as well as shorter fragments including tRF-5 (from the 5′ end), tRF-3 (from the 3′ end), and i-tRF (from internal regions) (20). MicroRNAs (miRNAs), well-established post-transcriptional regulators, have been extensively studied in sepsis and shown to modulate immune cell function, inflammatory cascades, and organ dysfunction (21–23). The stability of small RNAs in circulation, their tissue-specific expression patterns, and their functional involvement in sepsis-related pathways make them particularly attractive as both diagnostic biomarkers and potential therapeutic targets (24–26). Furthermore, advances in high-throughput sequencing technologies and bioinformatics approaches have enabled comprehensive profiling of circulating small RNA populations (27), opening new avenues for biomarker discovery and personalized medicine approaches in sepsis management.

In this study, we aimed to investigate the potential of circulating small RNAs as novel prognostic biomarkers for sepsis outcomes through comprehensive profiling of tsRNAs and miRNAs in serum samples from sepsis patients with different clinical trajectories. We employed an optimized small RNA detection protocol incorporating de-modification enzymes to enhance the capture of circulating tsRNAs and performed systematic comparative analysis between patients who recovered and those who died during their intensive care unit stay. Through this comprehensive molecular approach, we sought to develop novel biomarker signatures that could improve sepsis outcome prediction and contribute to more personalized and effective management strategies for critically ill patients.

## Materials and methods

### Study design and patient population

This prospective observational study was conducted at the intensive care unit (ICU) of the First Affiliated Hospital of Ningbo University from April 2022 to November 2022. The study protocol was approved by the institutional ethics committee Approval No.2021-R145, and written informed consent was obtained from all patients or their legal representatives. Adult patients (≥18 years) admitted to the ICU with a clinical diagnosis of sepsis according to the Sepsis-3 criteria were eligible for enrollment (28). Sepsis was defined as life-threatening organ dysfunction caused by a dysregulated host response to infection, identified by an increase in the Sequential Organ Failure Assessment (SOFA) score of 2 points or more. Exclusion criteria included: (1) age <18 years, (2) pregnancy, (3) active malignancy with ongoing chemotherapy or radiotherapy, (4) immunosuppressive therapy within the past 30 days, (5) previous organ transplantation, and (6) expected survival <24 h based on clinical assessment. A total of 26 patients were enrolled and stratified into two groups based on clinical outcomes: recovery group (survivors, *n* = 17) and death group (non-survivors, *n* = 9).

### Clinical data collection and laboratory measurements

Comprehensive clinical data were collected at ICU admission, including demographic characteristics (age, gender, body mass index), vital signs (temperature, heart rate, respiratory rate, blood pressure, oxygen saturation), and severity scores. The SOFA score and Acute Physiology and Chronic Health Evaluation II (APACHE II) score were calculated within 24 h of ICU admission according to standard protocols. Blood samples were collected within 6 h of ICU admission for routine laboratory analyses and small RNA profiling. Laboratory parameters included inflammatory biomarkers (procalcitonin, C-reactive protein, interferon-*γ*, interleukin-6, interleukin-10, tumor necrosis factor-*α*), organ function indicators (serum creatinine, blood urea nitrogen, alanine aminotransferase, aspartate aminotransferase, gamma-glutamyl transferase, total bilirubin), metabolic parameters (glucose), electrolyte balance (potassium, sodium, magnesium, calcium, phosphorus), and acid–base balance (pH, actual bicarbonate, standard bicarbonate). All laboratory measurements were performed using standard clinical chemistry analyzers according to manufacturer protocols.

### Serum sample collection and processing

Venous blood samples (5 mL) were collected in serum separator tubes and allowed to clot at room temperature for 30 min. Samples were centrifuged at 3,000 rpm for 10 min at 4 °C to separate serum. The serum was carefully transferred to RNase-free tubes, avoiding the buffy coat layer, and immediately stored at −80 °C until RNA extraction. To minimize hemolysis-related interference, samples with visible hemolysis were excluded from the analysis.

### RNA extraction and quality assessment

Total RNA was extracted from 200 μL of serum using the miRNeasy Serum/Plasma Kit (Qiagen, Hilden, Germany) according to the manufacturer’s instructions with modifications to enhance small RNA recovery. Briefly, serum samples were lysed with QIAzol lysis reagent, followed by chloroform extraction and isopropanol precipitation. The RNA pellet was washed with 75% ethanol and dissolved in RNase-free water. RNA concentration and purity were assessed using a NanoDrop 2000 spectrophotometer (Thermo Fisher Scientific, Waltham, MA, United States). RNA integrity was evaluated using an Agilent 2,100 Bioanalyzer (Agilent Technologies, Santa Clara, CA, United States) with the Small RNA Kit.

### Small RNA library preparation and sequencing

To comprehensively capture circulating small RNAs, particularly tRNA-derived fragments, an optimized library preparation protocol incorporating RNA modification removal was employed. The workflow included: (1) deacylation to remove amino acid modifications, (2) 3′-cyclic phosphate (3’-cP) removal using T4 polynucleotide kinase, (3) 5′-phosphate addition, and (4) demethylation using AlkB demethylase to remove methyl modifications that could interfere with adapter ligation. Small RNA libraries were constructed using the NEBNext Small RNA Library Prep Set for Illumina (New England Biolabs, Ipswich, MA, United States) following the manufacturer’s protocol. Briefly, 3′ and 5′ adapters were ligated to the processed RNA, followed by reverse transcription and PCR amplification. Library quality was assessed using an Agilent 2,100 Bioanalyzer, and quantification was performed using the KAPA Library Quantification Kit. The final libraries were pooled and sequenced (2 × 150 bp) using the Illumina NovaSeq 6,000 platform (Illumina, San Diego, CA, United States).

### Bioinformatics analysis and small RNA annotation

Raw sequencing reads were processed using a standardized bioinformatics pipeline. Quality control was performed using FastQC v0.11.9, and adapter sequences were trimmed using Cutadapt v3.4. Reads shorter than 15 nucleotides or longer than 50 nucleotides were filtered out. Clean reads were mapped to the human reference genome (GRCh38) using Bowtie2 v2.4.4 with parameters optimized for small RNA alignment. Small RNA annotation was performed using multiple databases: miRBase v22 for miRNA annotation, GtRNAdb for tRNA sequences, and custom databases for tsRNA classification. Only small RNAs that were expressed in at least 70% of patients were retained for subsequent analysis. Read counts were normalized using the trimmed mean of M-values (TMM) method implemented in edgeR v3.36.0.

### KEGG functional enrichment analysis

To investigate the functional significance of the identified small RNA biomarkers, we performed KEGG pathway enrichment analysis on their predicted target genes. For miRNA target gene prediction, we employed two computational algorithms: TargetScan (version 5.0) and Miranda (version 3.3a) through the OmicStudio platform,^1^ with filtering thresholds set at TargetScan_score ≥ 50 and miranda_Energy < −20. The predicted target genes from both algorithms were merged and overlapping targets were identified to ensure prediction reliability. For tsRNA target gene prediction, we utilized GSTAr (version 1.0) with MFEratio > 0.65 and AllenScore < 4, and PsRobot (version 1.2) with a scoring threshold < 2.5. Kyoto Encyclopedia of Genes and Genomes (KEGG) pathway enrichment analysis was conducted by mapping all predicted target genes to the KEGG database.^2^ Gene counts for each pathway were calculated, and hypergeometric testing was performed to identify KEGG pathways that were significantly enriched in the target genes compared to the genomic background.

### Statistical analysis

Statistical analyses were performed using R software v4.4 and GraphPad Prism v10.4. Continuous variables were expressed as mean ± standard deviation (SD) and compared using Student’s *t*-test or Mann–Whitney U test depending on data distribution. Categorical variables were presented as frequencies and percentages and analyzed using chi-square test. Principal component analysis (PCA) was performed to visualize overall expression patterns between groups. Differential expression analysis was conducted using DESeq2 v1.34.0 with the following criteria: fold change > 1.5 or <0.5 and *p*-value < 0.05. Receiver operating characteristic (ROC) curve analysis was performed to evaluate the diagnostic performance of individual biomarkers and combined panels. The area under the curve (AUC) was calculated with 95% confidence intervals. For biomarker panel construction, we employed a systematic two-step feature selection strategy. First, from the pool of significantly differentially expressed small RNAs identified by DESeq2, we performed individual ROC curve analysis for each small RNA to evaluate their discriminatory performance. Second, for combined panel construction, we selected the top three performing individual biomarkers based purely on their individual AUC values for each small RNA category (tsRNAs and miRNAs separately). Statistical significance was set at *p* < 0.05 for all analyses.

## Results

### Baseline characteristics and initial clinical presentation

This study enrolled 26 sepsis patients admitted to the intensive care unit (ICU), who were subsequently stratified into two groups based on clinical outcomes: a recovery group (*n* = 17) and a death group (*n* = 9). Baseline demographic characteristics showed no significant differences between groups, including age, gender distribution, and body mass index (Supplementary Table S1). Initial vital signs at ICU admission were also comparable between groups: temperature, respiratory rate, oxygenation parameters, heart rate, and proportion of patients with low systolic blood pressure (Supplementary Table S2). These findings indicate that demographic factors and initial clinical presentation were not predictive of sepsis outcomes.

### Severity scores and organ dysfunction assessment

Despite comparable initial presentations, standardized severity scoring systems revealed critical prognostic differences (Supplementary Table S3). The Sequential Organ Failure Assessment (SOFA) score was significantly higher in the death group compared to the recovery group, suggesting that the degree of multi-organ dysfunction was strongly associated with mortality risk. However, the Acute Physiology and Chronic Health Evaluation II (APACHE II) scores showed no significant difference between groups, suggesting that organ-specific dysfunction assessment provides superior prognostic discrimination compared to composite severity scores incorporating chronic health status and age factors.

### Laboratory parameters and biomarker profiles

**Figure 1:**
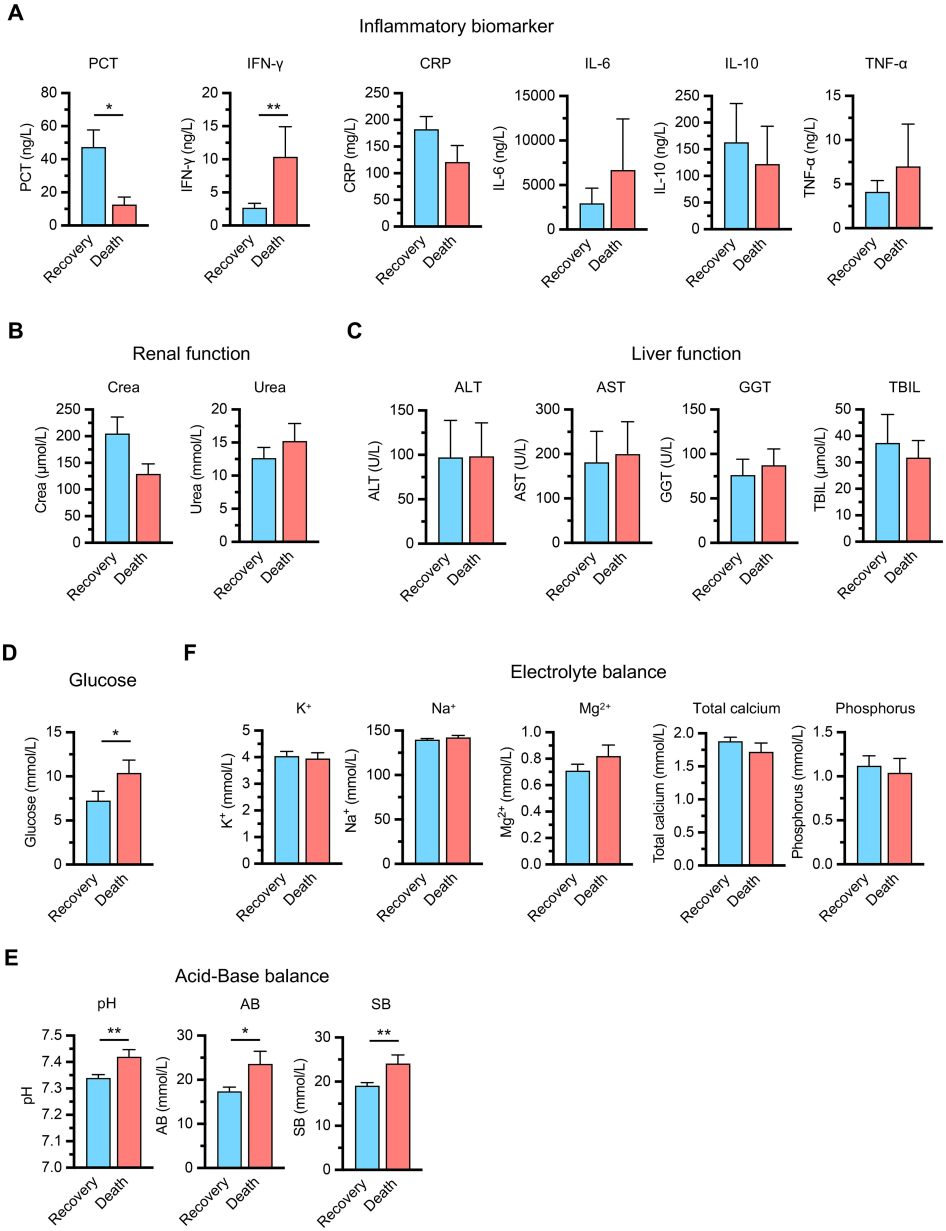
Clinical laboratory parameters comparison between recovery (n = 17) and death (n = 9) groups in sepsis patients. **(A)** Inflammatory biomarkers including procalcitonin (PCT), interferon-*γ* (IFN-γ), C-reactive protein (CRP), interleukin-6 (IL-6), interleukin-10 (IL-10), and tumor necrosis factor-*α* (TNF-α). **(B)** Renal function markers including serum creatinine (Crea) and blood urea nitrogen (Urea). **(C)** Liver function indicators including alanine aminotransferase (ALT), aspartate aminotransferase (AST), gamma-glutamyl transferase (GGT), and total bilirubin (TBIL). **(D)** Metabolic parameters (glucose). **(E)** Acid–base balance parameters including pH, actual bicarbonate (AB), and standard bicarbonate (SB). **(F)** Electrolyte balance including potassium (K^+^), sodium (Na^+^), magnesium (Mg^2+^), total calcium, and phosphorus. Data are presented as mean ± SEM. *p < 0.05, **p < 0.01 compared between groups (Student’s *t*-test for CRP, Urea, K^+^, Na^+^, Mg^2+^, phosphorus, pH, AB, SB, and the Mann–Whitney U test for the remaining parameters).

To further characterize the pathophysiological differences between groups, we analyzed comprehensive laboratory parameters including inflammatory markers, organ function indicators, metabolic parameters, acid–base balance, and electrolyte levels (Figure 1). The inflammatory biomarker profile demonstrated complex patterns that distinguished clinical outcomes. Procalcitonin (PCT) levels were higher in the recovery group compared to the death group. Conversely, interferon-*γ* (IFN-γ) levels were significantly elevated in the death group, potentially indicating maladaptive immune hyperactivation contributing to organ dysfunction. Other inflammatory mediators, including C-reactive protein (CRP), interleukin-6 (IL-6), interleukin-10 (IL-10) and tumor necrosis factor-*α* (TNF-α), showed comparable levels between groups (Figure 1A).

Organ function assessment revealed no significant differences between groups, with renal function markers including serum creatinine (Crea) and blood urea nitrogen (Urea) showing similar patterns across both groups (Figure 1B). Hepatic function indicators, including alanine aminotransferase (ALT), aspartate aminotransferase (AST), gamma-glutamyl transferase (GGT), and total bilirubin levels (TBIL), were comparable between groups (Figure 1C). However, metabolic parameters demonstrated critical prognostic differences, as the death group exhibited elevated glucose levels, reflecting impaired cellular metabolism associated with poor outcomes (Figure 1D). Acid–base analysis revealed unexpected patterns, with the death group showing elevated pH and bicarbonate levels, suggesting metabolic alkalosis in non-survivors that likely represents failed compensatory mechanisms or distinct pathophysiological trajectories (Figure 1E). Electrolyte parameters including potassium, sodium, magnesium, total calcium and phosphorus levels were comparable between groups (Figure 1F). These findings indicate that specific metabolic and immune markers, particularly PCT, IFN-*γ*, glucose, and acid–base parameters, rather than traditional organ function indicators, are primary determinants of sepsis outcomes.

### Small RNA profiling in sepsis patients’ serum

**Figure 2:**
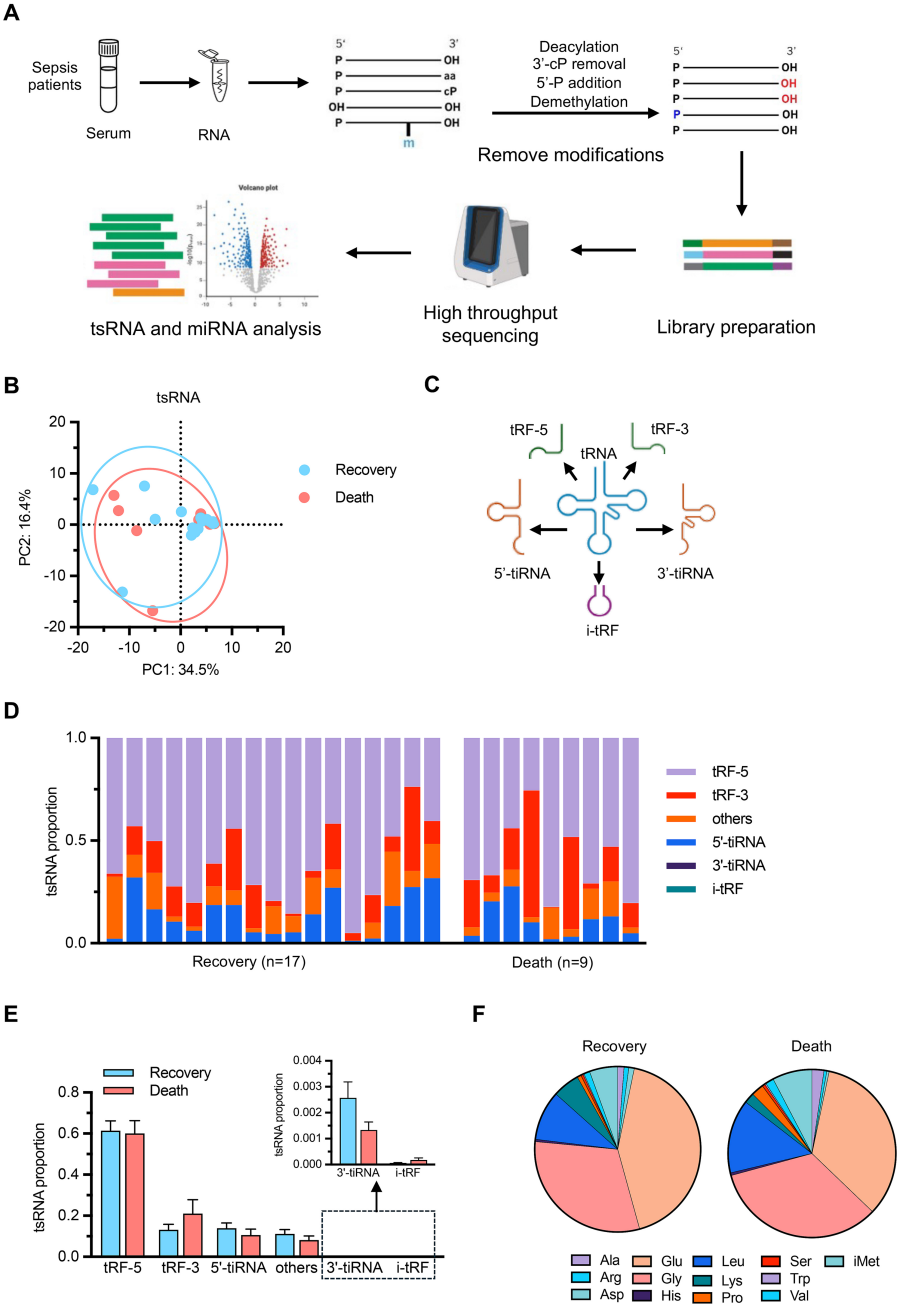
tRNA-derived small RNA (tsRNA) profiling **(A)** Schematic workflow of the optimized small RNA detection protocol. Serum samples from sepsis patients underwent RNA extraction followed by deacylation, 3’-cP removal, 5’-P addition, and demethylation to remove RNA modifications. Library preparation was performed followed by high-throughput sequencing and bioinformatics analysis of tsRNAs and miRNAs. **(B)** PCA of tsRNA expression profiles. *n* = 17 for recovery group, *n* = 9 for death group. **(C)** Schematic illustration of tsRNA classification categories including 5’-tiRNAs, 3’-tiRNAs, tRF-5, tRF-3, and i-tRF. **(D)** Individual sample distribution of tsRNA subtypes. **(E)** Statistical comparison of tsRNA subtype abundance between groups with detailed view of low-abundance categories (3’-tiRNA and i-tRF). **(F)** Pie charts showing the distribution of tsRNAs derived from different parental tRNA amino acid species in recovery and death groups. Data are presented as mean ± SEM. No statistically significant differences were observed between groups by student’s *t*-test in **(E)**.

To further investigate the molecular biomarkers underlying sepsis outcomes, we explored the small RNA profiles in patients’ serum samples using an optimized detection protocol. The experimental workflow incorporated de-modification enzymes to enhance the comprehensive capture of the circulating small RNA landscape, particularly tRNA-derived small RNAs (tsRNAs) (Figure 2A). Principal component analysis revealed no significant differences in tsRNA composition between the recovery and death groups (Figure 2B). We also classified tsRNAs into several functional categories, including 5’-tiRNAs, 3’-tiRNAs, tRF-3, tRF-5, and i-tRF, as well as unclassified fragments (others) (Figure 2C) (20). Statistical analysis of tsRNA subtype abundance showed no significant differences between groups (Figures 2D,E), and analysis of tsRNAs derived from different parental tRNA species similarly revealed no significant intergroup variations (Figure 2F). These findings reveal that the overall composition of serum tsRNAs do not undergo significant alterations in sepsis patients with different clinical outcomes.

**Figure 3:**
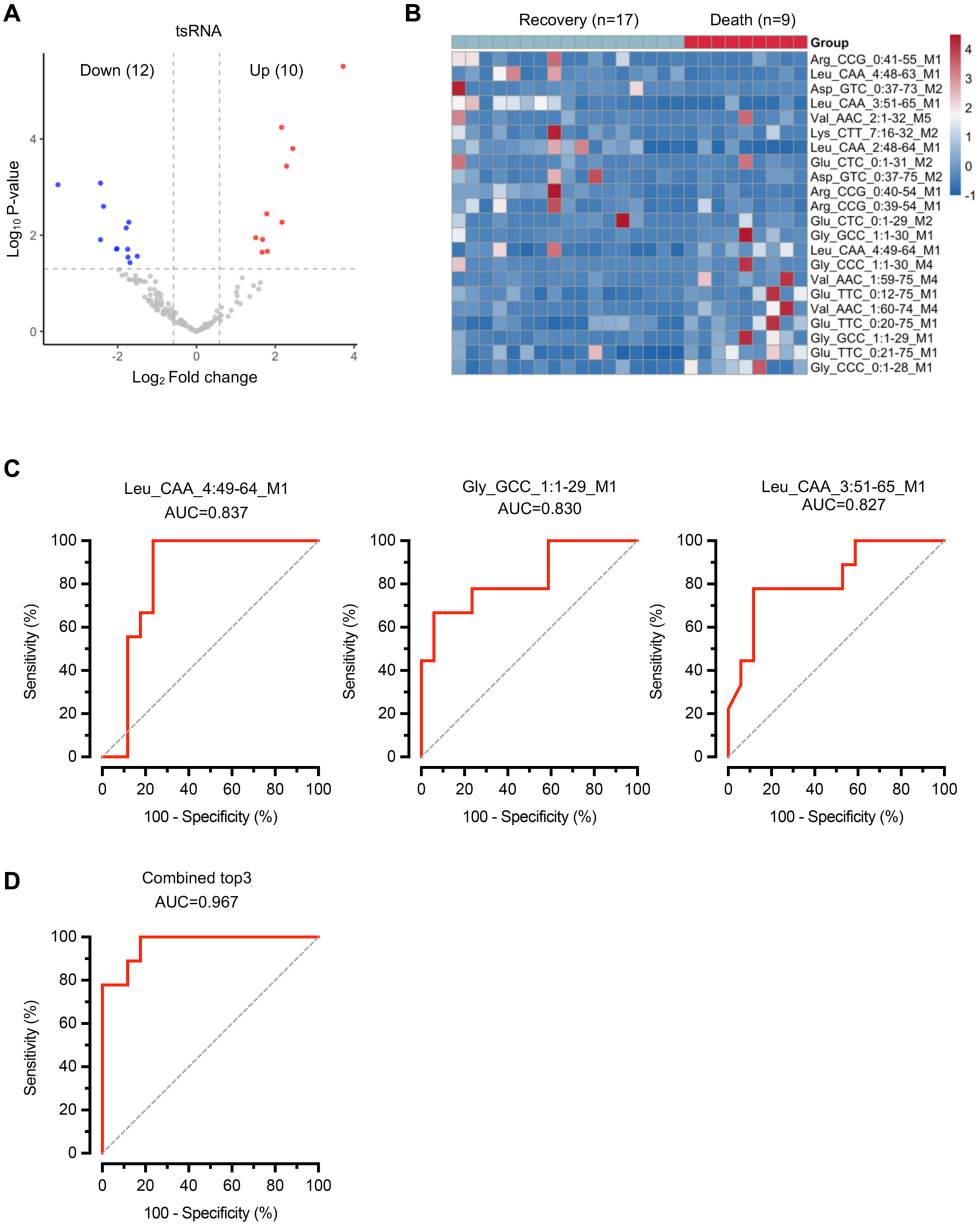
Differential expression analysis of tsRNAs reveals specific biomarkers for sepsis outcome prediction. **(A)** Volcano plot showing differentially expressed tsRNAs. Red dots represent upregulated tsRNAs and blue dots represent downregulated tsRNAs in the death group compared to the recovery group. Gray dots indicate non-significantly altered tsRNAs. **(B)** Heatmap displaying the expression patterns of 22 significantly differentially expressed tsRNAs across all samples. *n* = 17 for recovery group, *n* = 9 for death group. **(C)** ROC curves for the top three performing individual tsRNA biomarkers. **(D)** ROC curve for the combined biomarker panel of the top three tsRNAs. Diagonal dashed lines represent random classification (AUC = 0.5).

Subsequently, we performed comparative analysis to identify differentially expressed tsRNAs between the two groups with distinct clinical outcomes. Differential expression analysis revealed 22 significantly altered tsRNAs, with 12 downregulated and 10 upregulated in the death group compared to the recovery group (Figure 3A; Supplementary Table S4). The heatmap revealed distinct expression patterns, with several tsRNAs showing consistent upregulation or downregulation across samples within each group (Figure 3B). To evaluate the diagnostic potential of these differentially expressed tsRNAs, we conducted receiver operating characteristic (ROC) curve analysis for individual biomarkers. The top three performing tsRNAs demonstrated substantial discriminatory power: tsRNA-Leu_CAA_4:49-64_M1 (AUC = 0.837, 95% CI: 0.674–0.999), tsRNA-Gly_GCC_1:1-29_M1 (AUC = 0.830, 95% CI: 0.654–1.000), and tsRNA-Leu_CAA_3:51-65_M1 (AUC = 0.827, 95% CI: 0.655–0.999) (Figure 3C). Notably, when these three tsRNAs were combined into a composite biomarker panel, the diagnostic performance was markedly enhanced, achieving an AUC of 0.967 (95% CI: 0.908–1.000) (Figure 3D). This exceptional discriminatory capacity suggests that the combined tsRNA signature holds significant clinical potential as a prognostic biomarker for predicting sepsis outcomes. These findings suggest that while individual tsRNAs show promising diagnostic value, their combination substantially improves the ability to distinguish between sepsis patients with different clinical trajectories.

**Figure 4:**
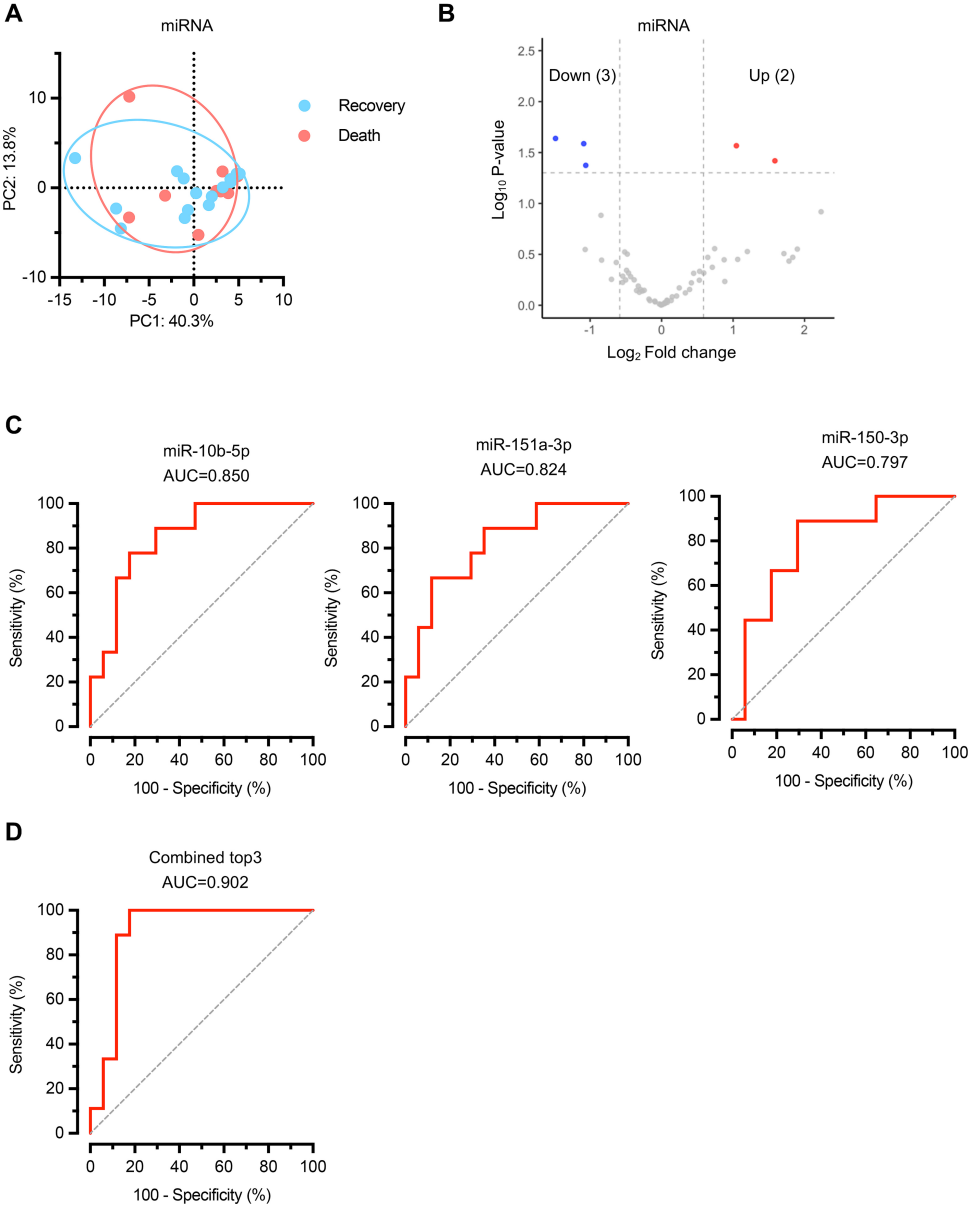
MicroRNA expression profiling identifies specific biomarkers for sepsis outcome prediction. **(A)** PCA of miRNA expression profiles. *n* = 17 for recovery group, *n* = 9 for death group. **(B)** Volcano plot displaying differentially expressed miRNAs between recovery and death groups. Red dots represent upregulated miRNAs and blue dots represent downregulated miRNAs in the death group compared to the recovery group. Gray dots indicate non-significantly altered miRNAs. **(C)** ROC curves for the top three performing individual miRNA biomarkers. **(D)** ROC curve for the combined biomarker panel of the top three miRNAs. Diagonal dashed lines represent random classification (AUC = 0.5).

We also examined miRNA expression profiles between the recovery and death groups. Principal component analysis of miRNA expression data revealed no significant differences in overall miRNA composition between the two groups (Figure 4A), indicating that global miRNA profiles remained comparable regardless of clinical outcomes. Differential expression analysis identified 5 significantly altered miRNAs, with 3 downregulated and 2 upregulated in the death group compared to the recovery group (Figure 4B; Supplementary Table S5). Receiver operating characteristic (ROC) curve analysis was performed to assess the individual diagnostic potential of each differentially expressed miRNA (Figure 4C). The top three performing miRNAs demonstrated substantial discriminatory power: miR-10b-5p (AUC = 0.850, 95% CI: 0.701–0.998), miR-151a-3p (AUC = 0.824, 95% CI: 0.659–0.988), and miR-150-3p (AUC = 0.797, 95% CI: 0.619–0.976). When these three miRNAs were combined into a composite biomarker panel, the diagnostic performance was significantly enhanced, achieving an AUC of 0.902 (95% CI: 0.776–1.000) (Figure 4D). These findings demonstrate that specific miRNAs can also serve as valuable predictive biomarkers for sepsis outcomes.

### Functional enrichment analysis of small RNA biomarkers

**Figure 5:**
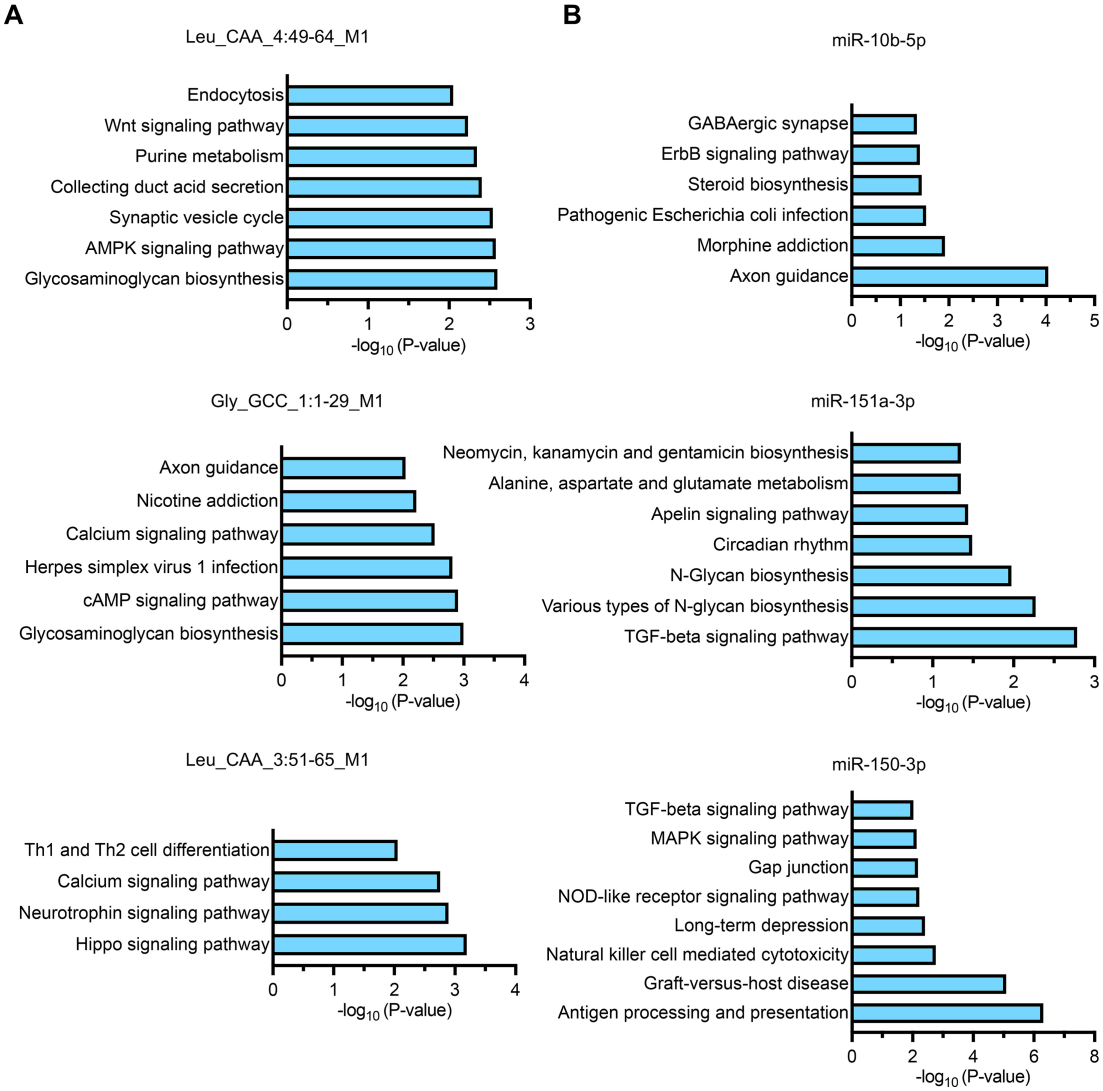
KEGG pathway enrichment analysis of target genes for individual small RNA biomarkers. **(A,B)** Bar charts showing the top significantly enriched pathways for each biomarker based on target gene analysis. The x-axis represents the -log10(*p*-value) indicating the statistical significance of pathway enrichment.

The analysis revealed distinct pathway specializations for each biomarker: tsRNA-Leu_CAA_4:49-64_M1 was primarily enriched in glycosaminoglycan biosynthesis, AMPK signaling pathway, and synaptic vesicle cycle; tsRNA-Gly_GCC_1:1-29_M1 demonstrated enrichment in glycosaminoglycan biosynthesis, cAMP signaling pathway, and herpes simplex virus 1 infection pathways; tsRNA-Leu_CAA_3:51-65_M1 was predominantly enriched in Hippo signaling pathway, neurotrophin signaling pathway, and calcium signaling pathway (Figure 5A). Among miRNA biomarkers, miR-10b-5p showed primary enrichment in axon guidance, morphine addiction, and pathogenic *Escherichia coli* infection pathways; miR-151a-3p was enriched in TGF-beta signaling pathway, various types of N-glycan biosynthesis, and N-glycan biosynthesis; while miR-150-3p demonstrated enrichment in antigen processing and presentation, graft-versus-host disease, and natural killer cell-mediated cytotoxicity pathways (Figure 5B). These functional enrichment patterns reveal that our identified small RNA biomarkers collectively target diverse biological processes essential for sepsis pathophysiology, including metabolic regulation, immune responses, cellular signaling, and tissue homeostasis, providing mechanistic insights into their roles in determining sepsis outcomes and supporting their utility as both prognostic indicators and potential therapeutic targets.

## Discussion

This study provides novel insights into the molecular determinants of sepsis outcomes through comprehensive analysis of clinical parameters and circulating small RNA profiles. Our findings reveal that while traditional demographic factors and initial clinical presentations show limited prognostic value, specific metabolic and immune markers, particularly PCT, IFN-*γ*, glucose levels, and acid–base parameters, demonstrate significant associations with sepsis outcomes. Most importantly, we identified distinct circulating small RNA signatures, including 22 differentially expressed tsRNAs and 5 differentially expressed miRNAs, that exhibit exceptional diagnostic performance for predicting sepsis mortality. The combination of top-performing tsRNAs achieved an AUC of 0.967, while the miRNA panel reached an AUC of 0.902, substantially outperforming individual biomarkers and suggesting significant clinical potential for molecular-based prognostic stratification in sepsis patients.

Traditional clinical parameters and biomarkers have shown limited efficacy in accurately predicting sepsis outcomes, highlighting the urgent need for novel prognostic indicators (6). While the SOFA score demonstrated prognostic value in our cohort, consistent with its established role in assessing organ dysfunction severity (11), the majority of conventional markers including APACHE II scores, standard inflammatory mediators (CRP, IL-6, IL-10, TNF-*α*), and organ function parameters showed no significant differences between outcome groups. PCT levels were elevated in the recovery group compared to the death group, a finding consistent with previous studies reporting that initial PCT levels may not serve as optimal prognostic indicators and that PCT dynamics rather than absolute values may be more informative for outcome prediction (29–31). This observation may reflect several factors: robust early inflammatory response capability in survivors that facilitates pathogen clearance, different disease stages at admission reflecting sepsis heterogeneity, or complex immune dynamics involving simultaneous hyperinflammation and immunosuppression. Additionally, increased IFN-γ levels in the death group suggest dysregulated immune activation associated with adverse outcomes. These findings underscore the limitations of current prognostic tools and emphasize the critical need for innovative biomarkers that can provide more accurate and early prediction of sepsis outcomes to guide clinical decision-making and therapeutic interventions.

Our study demonstrates for the first time that circulating small RNAs, particularly tsRNAs and miRNAs, represent promising novel biomarkers for sepsis outcome prediction. The identification of specific tsRNA and miRNA signatures with exceptional discriminatory power represents a significant advancement in sepsis biomarker research. The performance of combined biomarker panels (AUC = 0.967 for tsRNAs and 0.902 for miRNAs) compared to individual markers suggests that small RNA signatures capture complex pathophysiological processes underlying sepsis pathogenesis and progression. While the PCA showed limited separation between outcome groups, this apparent contradiction with the high discriminatory power of individual biomarkers reflects the fact that effective biomarkers often represent subtle but highly specific molecular changes that may not constitute the primary sources of variance captured by unsupervised dimensionality reduction techniques. This pattern emphasizes that sepsis outcome prediction may depend on precise regulatory mechanisms rather than global expression pattern changes.

Our functional enrichment analysis aligns with emerging evidence that small RNAs play crucial roles in immune regulation, inflammatory responses, and cellular stress responses during sepsis. Recent mechanistic studies have further validated the functional importance of tsRNAs in sepsis-related pathophysiology, particularly in septic cardiomyopathy, where 158 differentially expressed tsRNAs were identified in experimental models, with the majority (101 tsRNAs) being upregulated (32). Importantly, these tsRNAs demonstrated protective effects against cardiomyocyte death through modulation of cellular processes and the Wnt signaling pathway, with interference of angiogenin (ANG) (33), a key nuclease responsible for tsRNA production, resulting in increased cardiomyocyte death and reduced cell viability (32). This mechanistic evidence supports our findings that tsRNA alterations are not merely passive biomarkers but may actively participate in sepsis pathophysiology and organ protection mechanisms. The stability of small RNAs in circulation, their tissue-specific expression patterns, and their functional roles in gene regulation make them particularly attractive as both biomarkers and potential therapeutic targets (34). The high diagnostic accuracy achieved by these molecular signatures suggests their potential for clinical translation to improve sepsis management and patient stratification.

Several limitations should be acknowledged in interpreting our findings. First, the relatively small sample size (*n* = 26) may limit the generalizability of our results and the statistical power for detecting smaller effect sizes. This limited sample size poses a substantial risk of overfitting, particularly in our ROC analyses and logistic regression models that combine multiple biomarkers. The small number of outcome events may lead to unstable model parameters and overoptimistic performance estimates. Validation in larger, multicenter cohorts is essential to confirm the robustness and clinical utility of these small RNA biomarkers.

Second, our single time-point sampling approach (within 6 h of ICU admission) represents a significant limitation that may affect the clinical interpretation and applicability of our identified biomarkers. Small RNA levels may fluctuate dynamically throughout the sepsis course (21, 35), and our early sampling strategy captures only the initial molecular signature, potentially missing crucial temporal changes that occur during disease progression. While this early sampling approach remains clinically relevant for timely risk stratification, the dynamic nature of biomarker expression during sepsis progression may affect the generalizability of our findings across different disease stages.

Third, the observational nature of our study precludes determination of causal relationships between small RNA alterations and sepsis outcomes. Although our functional enrichment analysis provides mechanistic insights into potential pathways involved, the lack of functional validation experiments limits our understanding of the biological significance of the differentially expressed small RNAs. Future mechanistic studies are needed to determine whether these molecules represent mere biomarkers or active participants in disease progression.

Finally, the heterogeneity of sepsis etiology and patient populations may influence biomarker performance, necessitating validation across diverse clinical settings and patient subgroups before clinical implementation. Future longitudinal validation studies examining temporal biomarker patterns throughout sepsis progression will be essential to fully understand the clinical utility of these molecular signatures and address these methodological limitations.

## Data Availability

The datasets presented in this study can be found in online repositories. The names of the repository/repositories and accession number(s) can be found in the article/Supplementary material.

## Footnotes

^1^ https://www.omicstudio.cn/tool

^2^ https://www.kegg.jp/
