# Supplementary material for: Serum small RNA profiling identifies prognostic biomarkers for sepsis mortality prediction
Source: Front Med (Lausanne). 2025 Sep 29;12:1665726. doi: 10.3389/fmed.2025.1665726 (PMC12515841; doi:10.3389/fmed.2025.1665726)
Supplement: Supplementary file 1 [file Table_1.docx]

**Table S1 Baseline demographic characteristics of sepsis patients**

|  | **Recovery Group (n=17)** | **Death Group (n=9)** | ***P* value** |
| --- | --- | --- | --- |
| Age (year) | 67.41±16.66 | 67.67±13.40 | 0.9684 |
| Sex, Male n (%) | 13 (76.47%) | 7 (77.78%) | 1.0000 |
| BMI | 23.70±4.02 | 22.40±4.73 | 0.4663 |

BMI: body mass index.

**Table S2 Initial vital signs and hemodynamic parameters at ICU admission**

|  | **Recovery Group (n=17)** | **Death Group (n=9)** | ***P* value** |
| --- | --- | --- | --- |
| Temperature (°C) | 37.01 ± 0.96 | 37.36 ± 1.07 | 0.4044 |
| Respiratory rate (breaths/min) | 20.41 ± 7.20 | 16.78 ± 5.21 | 0.2441 |
| PaO_2_/SaO_2_ (mmHg) | 114.49 ± 39.08 | 128.18 ± 74.60 | 0.7916 |
| Heart rate (beats/min) | 111.41 ± 29.77 | 91.89 ± 21.18 | 0.0946 |
| Low systolic blood pressure **^a,b^** (mmHg) | 11 (64.71%) | 4 (44.44%) | 0.4185 |

^a^ Systolic blood pressure was ≤100 mmHg.

^b^ Defined by “The third international consensus definitions for sepsis and septic Shock (Sepsis 3.0)” and “Chinese expert consensus on the diagnosis and treatment of sepsis-induced coagulopathy (2024 edition)”.

PaO_2_: partial pressure of oxygen in arterial blood; SaO_2_: oxygen saturation in arterial blood.

**Table S3 Disease severity scores**

|  | **Recovery Group (n=17)** | **Death Group (n=9)** | ***P* value** |
| --- | --- | --- | --- |
| APACHE II score (points) | 23.53 ± 6.45 | 25.11 ± 6.94 | 0.5675 |
| SOFA score (points) | 10.18 ± 4.32 | 14.44 ± 3.32 | **0.0165** |

APACHE II: Acute Physiology and Chronic Health Evaluation II; SOFA: Sequential Organ Failure Assessment.

**Table S4 Differentially expressed tsRNAs**

| **tsRNA name** | **log_2_FoldChange** | ***P* value** | **AUC** |
| --- | --- | --- | --- |
| Leu_CAA_4:49-64_M1 | 1.6652 | 0.0226 | 0.8366 |
| Gly_GCC_1:1-29_M1 | 2.2849 | 0.0004 | 0.8301 |
| Leu_CAA_3:51-65_M1 | -2.3595 | 0.0025 | 0.8268 |
| Glu_TTC_0:21-75_M1 | 2.4440 | 0.0002 | 0.8235 |
| Gly_GCC_1:1-30_M1 | 1.5018 | 0.0113 | 0.8170 |
| Leu_CAA_2:48-64_M1 | -1.7833 | 0.0071 | 0.8170 |
| Gly_CCC_0:1-28_M1 | 3.7192 | 0.0425 | 0.8072 |
| Val_AAC_1:60-74_M4 | 2.1568 | 0.0006 | 0.8039 |
| Arg_CCG_0:40-54_M1 | -1.7164 | 0.0054 | 0.7908 |
| Glu_TTC_0:20-75_M1 | 2.1681 | 0.0054 | 0.7712 |
| Glu_TTC_0:12-75_M1 | 1.7991 | 0.0216 | 0.7451 |
| Leu_CAA_4:48-63_M1 | -2.4360 | 0.0008 | 0.7320 |
| Val_AAC_1:59-75_M4 | 1.7817 | 0.0036 | 0.7320 |
| Asp_GTC_0:37-73_M2 | -1.7384 | 0.0287 | 0.6928 |
| Gly_CCC_1:1-30_M4 | 1.6759 | 0.0123 | 0.6209 |
| Asp_GTC_0:37-75_M2 | -2.4360 | 0.0124 | 0.6078 |
| Lys_CTT_7:16-32_M2 | -2.0193 | 0.0190 | 0.5882 |
| Arg_CCG_0:41-55_M1 | -3.5163 | 0.0009 | 0.5752 |
| Val_AAC_2:1-32_M5 | -2.0311 | 0.0194 | 0.5752 |
| Glu_CTC_0:1-31_M2 | -1.7456 | 0.0196 | 0.5621 |
| Glu_CTC_0:1-29_M2 | -1.5025 | 0.0273 | 0.5359 |
| Arg_CCG_0:39-54_M1 | -1.6876 | 0.0371 | 0.3856 |

**Table S5 Differentially expressed miRNAs**

| **miRNA name** | **log2FoldChange** | ***P* value** | **AUC** |
| --- | --- | --- | --- |
| miR-10b-5p | -1.4836 | 0.0230 | 0.8497 |
| miR-151a-3p | 1.0460 | 0.0271 | 0.8235 |
| miR-150-3p | 1.5852 | 0.0381 | 0.7974 |
| let-7b-5p | -1.0686 | 0.0466 | 0.7582 |
| miR-139-3p | -0.8881 | 0.0499 | 0.7222 |
